# Efficient feature selection and classification for microarray data

**DOI:** 10.1371/journal.pone.0202167

**Published:** 2018-08-20

**Authors:** Zifa Li, Weibo Xie, Tao Liu

**Affiliations:** Department of Computer Science and Technology, Huaqiao University, Xiamen, Fujian, China; Northeast Normal University, CHINA

## Abstract

Feature selection and classification are the main topics in microarray data analysis. Although many feature selection methods have been proposed and developed in this field, SVM-RFE (Support Vector Machine based on Recursive Feature Elimination) is proved as one of the best feature selection methods, which ranks the features (genes) by training support vector machine classification model and selects key genes combining with recursive feature elimination strategy. The principal drawback of SVM-RFE is the huge time consumption. To overcome this limitation, we introduce a more efficient implementation of linear support vector machines and improve the recursive feature elimination strategy and then combine them together to select informative genes. Besides, we propose a simple resampling method to preprocess the datasets, which makes the information distribution of different kinds of samples balanced and the classification results more credible. Moreover, the applicability of four common classifiers is also studied in this paper. Extensive experiments are conducted on six most frequently used microarray datasets in this field, and the results show that the proposed methods have not only reduced the time consumption greatly but also obtained comparable classification performance.

## 1. Introduction

The invention of DNA microarray technology has spawned massive gene expression microarray data, which brings a new way for the gene-related studies, mainly gene recognition and disease diagnosis [1]. Over the decades, however, the characteristics of these data have remained almost unchanged. Among these characteristics, small sample size, high dimensions and class imbalance are the most typical issues to overcome [2]. Gene recognition is to find the genes that strongly associated with specific diseases, so it is actually a feature selection task. The quality of feature selection is usually evaluated by observing the classification performance. So, the classification plays an important role in gene recognition tasks. Disease diagnosis is essentially a classification task. For a classification task, the small size with a large number of features of the training dataset can easily lead to faulty generalization ability of the classification model [3]. Considering the characteristics of microarray data with small sample size and high dimensionality, it is necessary to reduce the dimensions before the classification. Feature selection is currently a good choice for dimensionality reduction for microarray data. Accordingly, a powerful feature selection method and appropriate classifier become indispensable whether for gene recognition or disease diagnosis for microarray data. On the other hand, a serious class imbalance problem can lead to the classification results that are not convincing enough [4]. To solve this problem, an effective resampling method is also a need.

Small sample size with high dimensionality is the main challenge of microarray data analysis, and class imbalance makes this case worse. Class imbalance refers to the large gap in the number of samples belonging to different classes, which often results in an unreliable classification result. For example, if given a test set containing two classes of samples, in which the sample number of A is two times that of the other B. In this case, if we predict all the samples in test set as A, then we would get the accuracy 66.67% which is much bigger than 50%. This is very unreasonable. That is to say, class imbalance can lower the credibility of classification accuracy. In addition to this, ranked features with imbalance dataset are also problematic because the information is unevenly distributed in training set. Therefore, various resampling technologies emerge to alleviate this issue [4–10]. Among them, the traditional methods are under-sampling and over-sampling. The principle of these methods is either to randomly delete samples from the major class or to randomly select samples from the minority one and then replicate them, which results in either loss of information or overfitting [10]. Later, a very popular resampling method known as SMOTE [4] is proposed. This method has been proved effective, so many improved versions are put forward one after another [5]. An obvious characteristic of SMOTE is that the values of the generated samples are synthesized. We therefore insist that these methods like SMOTE are not suitable for microarray data, especially when the goal is gene recognition because each value of microarray data is a product of gene expression which is biological. In recent years, ensemble methods [6–9] have received significant attention, and the results prove that they are competitive. However, these methods based on the ensemble are too complex for microarray data because these datasets generally have small sample sizes. In order to get more reliable feature ranking and more credible classification accuracy, we propose a simple but effective resampling method, which is especially suitable for microarray data, to preprocess the datasets. For the rare class, the proposed method in this paper does not randomly select samples but randomly select the feature value, and then construct a new sample with these selected values. The experimental results fully verify the proposed method.

Feature selection methods have received significant attention in the last decades, some of which emerge as new algorithms while some others merge as variants [1, 2]. For a long time, SVM (Support Vector Machine) has attracted many researchers’ interests because of its competitive performance in classification and inherent feature selection capability. Guyou et al. published their famous paper in the journal Machine Learning in 2002 [11]. In this paper, they proposed a novel feature selection method called SVM-RFE, in which they make best use of the capabilities of SVM mentioned above and recursively delete one feature that is the least important from the ranked list until the number of the remained features meets requirements. The results are very good, and this method is quickly taken as a benchmark feature selection algorithm in subsequent studies. One of the disadvantages of SVM-RFE is that it does not take into account the correlation probably hidden between features during the feature selection process. To solve this problem, Mundra et al. [12] developed a hybrid method which combined the mRMR [13] and SVM-RFE to select the more meaningful genes. Yoon S et al. [14] proposed a variant of SVM-RFE based on mutual information. These methods try to make up for the shortage of SVM-RFE in lack of considering the correlation between features and achieve comparable results. Another drawback of SVM-RFE is that the process of feature selection with SVM-RFE is extremely time-consuming, which attracts the interest of many researchers in this field. Tang et al. [15] proposed a two-stage SVM-RFE method to accelerate the process of feature selection. They paid much attention to the intrinsic complexity of gene expression data and tried to reduce the time consumption by setting different filter-out factors while selecting the most useful genes. Ding Y et al. [16] improved the RFE (Recursive Feature Elimination) by changing the number of features to delete during the iteration. Specifically, they delete 1 / (i+1) of the remaining features in the ith iteration. That is to say, if a microarray dataset has 20000 genes, 10000 genes will be deleted in the first iteration, and 5000 genes will be deleted in the second iteration. Obviously, this method is a little “too rude”. Although the procedure of feature selection is greatly accelerated, such a practice can easily affect the final feature selection quality. Besides, Yin J et al. [17] also developed RFE in their own ways. All of these methods achieve better performance and reduce time consumption to some extent. This paper is dedicated to thoroughly solve the huge time consumption of SVM-RFE under the premise of ensuring the quality of feature selection. Firstly, we improve RFE and propose a new version of RFE with variable step size. Step size means the number of features to eliminate in the process of iteration. More concretely, the step size decreases as the number of features to select reduces. When the latter reaches a certain point, the former keeps unchanged with one. In order to further speed up the process of feature selection, we introduce an efficient implementation of linear SVM to replace SVM and combine it with the improved RFE to conduct the procedure of feature selection just as SVM-RFE. The experiments show that we achieve a drastic reduction of time consumption along with the promising classification accuracy.

Classifiers are the core component of microarray data analysis. However, they are not given enough attention in the existing researches, which is not reasonable. Guyou et al. claim that the features selected matter more than the classifier used and the differences in classifiers had little effect on the results. Moreover, [11] just puts SVM, Golub et al. classifier, and Fisher’s linear discriminant together for experimental analysis, which is obviously insufficient. In [2], C4.5, naïve Bayes and SVM are used to conduct experiments on nine microarray datasets, and the results prove that SVM performs better. [1] draws a similar conclusion. The results in [18] show that SVM seems to be the most suitable classifier in this field without a doubt. However, most of these datasets they used have class imbalance problem which has negative influence on the classification accuracy to some extends. In this article, we choose six most frequently used binary microarray datasets and preprocess them with an oversampling method proposed in this article at first; and then select the meaningful features with the improved SVM-RFE; finally, we perform the classification tasks with k-Nearest Neighbors, Naïve Bayes, SVM and L2 regularized Logistic Regression [19], respectively. The results demonstrate that the classification accuracy comes from different classifiers is very different and SVM is not always the best choice which is different from the conclusions in [1, 2, 18].

The remainder of this article is organized as follows: In section 2, we introduce the methods presented in this paper successively, including random value-based oversampling, recursive feature elimination with variable step size and large-scale linear support vector machine. Section 3 presents the experiments, including the description of datasets, data preprocessing, parameters estimation, performances evaluation measures, experimental results and the discussion. Section 4 is the conclusion.

## 2. Methods

### 2.1 Random value-based oversampling

The values of microarray data are the results of genes expression which are biologically specific and should not be arbitrarily altered. The approach presented in this section aims to solve the class imbalance problem of microarray data while maximizing the biological significance without causing information loss and model overfitting.

Random value-based oversampling (RVOS) assumes that the samples with the same category label are subject to the same distribution. Under this assumption, we construct a data matrix with the minority class and choose one value randomly from each of column as the value of the corresponding position of the new sample; and then, save the current sample and repeat for k times to make the sample numbers of two classes are equal. Thus, we finally obtain k samples which are different from the source data but subject to the same distribution. The details of RVOS are shown in Table 1. The given data matrix X represents the microarray data of the minority class with samples at rows and genes at columns.

**Table 1:** The overview of RVOS.

Algorithm 1. Random value-based oversampling method for class imbalance issue		
1. Given data matrix **X**, and the number of new samples k;		
2. While k> = 1:		
	(1) For j = 1, 2, …, n (n denotes the column size of **X**):	
		• Randomly choose a value V from **X**_j_ (the jth column of **X**); • Save V to the corresponding position of the current new sample;
	(2) Save new sample to **X**;	
	(3) k = k– 1;	
3. Return **X.**		

### 2.2 Recursive feature elimination with variable step size

The RFE strategy introduced by Guyon et al. [11] is concretely an instance of backward feature elimination. Based on an external estimator that assigns weights to features, the goal of RFE is recursively eliminating the most unimportant feature or a subset of features arranged at the end. Firstly, the estimator is trained on the initial set of features and the weight is assigned to each one of them. Then, these absolute weights are sorted from large to small. Finally, the last feature or features are deleted. Repeat the procedure on the pruned set until the desired number of features to select is eventually reached.

The main shortcoming of RFE is the problem of tremendous time consumption, especially when the input dimensionality is extremely high. Therefore, it is indispensable to increase the step size so that the number of iterations will be decreased. However, some researchers state implicitly that a large step size would have a negative effect on the result of the feature selection, especially when the process of RFE is nearly completion [11]. Later, some other researchers insist that is not always true [15–17].

To reduce the time cost of RFE and minimize the adverse effect on feature selection simultaneously, we develop RFE strategy and propose an improved version called recursive feature elimination with variable step size (VSSRFE). Specifically, we firstly give the step size a large initial value, and then cut the value in half when the number of features to be eliminated has been reduced to half of its original size. Repeat the procedure until the step size is one. It can be deeply explained from two aspects: first, the step size varies from large to small and does not change every time. It depends on the update condition, update rule and the number of features to be removed. Second, the process of feature elimination is gradually refined from roughness. Generally, gene expression microarray data has a huge number of genes, and only a few of them are strongly related to the class labels. So, we have reason to believe that the relatively more genes excluded at first are very irrelevant with class labels. In other words, the later the gene is deleted the more significant it is. Therefore, in the earlier stage of feature selection, we can set a relatively large step size to reduce the number of iterations. At the later stage of the feature selection process, step size is reduced step by step and features are more carefully selected, thus ensuring the quality of selected features. This is the basis for us to improve RFE. In addition, we set the initial value of step size as a key parameter which relates to specific dataset. Table 2 shows the details.

**Table 2:** The overview of VSSRFE.

Algorithm 2. Recursive Feature Elimination with variable step size (VSSRFE)		
1. Given set of genes, **X**; labels of sample, **Y**; number of genes to select, n_selected; initial step size, s_initial		
2. Get total quantity of genes from **X**, n_total		
3. Temp = n_total; N = n_total; S = s_initial		
4. While N > n_selected:		
	(1) N = N–S;	
	(2) If temp / N = 2 and S > 1:	
		• Temp = N; • S = S / 2;
	(3) Train LLSVM with X and Y and get sorted weights vector **W**;	
	(4) Delete features according to **W** and S, and update **X**;	
5. Return **X.**		

### 2.3 Large-scale linear support vector machines

SVM is one of the best choices for feature selection and the most frequently employed classifier for microarray data. However, most of these SVMs are typical support vector machines, what is to say, these SVMs are all based on kernel techniques (generally, it is a linear kernel) and Lagrange dual solver (e.g. [11]). To accelerate the procedure of assigning weights, we introduce large-scale linear support vector machines (LLSVM) [20, 21] to replace SVM. LLSVM is a pure linear classifier designed for large-scale classification tasks such as text classification. Microarray data has super high dimensions just as text. So, it is worth trying to apply LLSVM to microarray data analysis. In addition to the feature weighting capability similar to SVM, LLSVM is a new implementation of SVM, which makes it exceptionally fast.

The objective function of LLSVM is defined as:

$$\underset{w}{\min}\;f\left(w\right)\equiv {\left\|w\right\|}_{1}+C\sum_{i\in I\left(w\right)}b_{i}{\left(w\right)}^{2}$$ (1)

Where

$$b_{i}\left(w\right)\equiv 1-y_{i}w^{T}x_{i}$$ (2)

$$I\left(w\right)\equiv \left\{i|b_{i}\left(w\right)>0\right\}$$ (3)

$x_{i}$ is the feature vector of the ith sample, $y_{i}$ is the corresponding label and w is the weight vector of features. So, LLSVM’s loss function is squared hinge which is L1 regularized. C > 0 is the penalty factor which determines how sparse w is. As C gets bigger, more weights of less important genes will be penalized to 0, i.e., wgets sparser. The final decision function has the same form just like other linear SVMs:

$$f\left(x^{*}\right)=sign\left(w\cdot x^{*}\right)$$ (4)

$x^{*}$ indicates the unknown feature vector of the sample to decide.

Unlike traditional SVMs which introduce Lagrange multipliers to solve a dual problem, yuan et al. in [20] applied cyclic coordinate descent algorithm to solve formula (1). From the current solution $w^{k}$, cyclic coordinate descent algorithm updates one variable to generate $w^{k,j}\in R^{n}$, j=1,…,n+1. J refers variable (feature) and k refers iteration. So $w^{k,1}=w^{k}$, $w^{k,n+1}=w^{k+1}$, and

$$w^{k,j}=\left[w_{1}^{k+1},\ldots ,w_{j-1}^{k+1},w_{j}^{k},\ldots ,w_{n}^{k}\right]\;\text{for}\;\text{j}=2,\ldots ,\text{n}.$$ (5)

To update $w^{k,j}$ to $w^{k,j+1}$, the following one-variable optimization problem is solved:

$$\underset{z}{\min}\;g_{j}\left(z\right)=|w_{j}+z|+L_{j}^{'}\left(0;w\right)z+\frac{1}{2}L_{j}^{''}\left(0;w\right)z^{2}+constant$$ (6)

Where

$$e_{j}={\left[0,\ldots ,0,1,0,\ldots ,0\right]}^{T}\in R^{n},$$ (7)

$$L_{j}\left(z;w\right)\equiv C\sum_{i\in I\left(w+ze_{j}\right)}b_{i}{\left(w+ze_{j}\right)}^{2},$$ (8)

And

$$L_{j}^{'}\left(0;w\right)=-2C\sum_{i\in I\left(w\right)}y_{i}x_{i}b_{i}\left(w\right),$$ (9)

$$L_{j}^{''}\left(0;w\right)=\max\left(2C\sum_{i\in I\left(w\right)}x_{i,j}^{2},10^{-12}\right),$$ (10)

Formula (6) is an approximate expression as $L_{j}\left(z;w\right)$ is not twice differentiable. Z is the change value of the j variable. If the solution of formula (6) is $z^{*}$, then we update the jth element by:

$$w_{j}^{k,j+1}=w_{j}^{w,j}+z^{*}$$ (11)

One iteration is completed when all variables have been updated. After m iterations, the result tends to be stable. Readers can find the details in [20, 21], and the framework of the algorithm is showed in Table 3.

**Table 3:** The overview of cyclic coordinate descent method for LLSVM.

Algorithm 3. Cyclic coordinate descent method for LLSVM		
1. Given $w^{1}$;		
2. For k = 1, 2, 3, … m:		
	(1) $w^{k,1}=w^{1}$;	
	(2) For j = 1, 2, …, n:	
		• Obtain Z^*^ by solving the sub-problem (6); • $w^{k,j+1}=w^{k,j}+{\text{z}}^{*}e_{j}$;
3. Return $w^{m+1}$.		

## 3. Experiments

This section focuses on the experimental verification of the methods proposed in this paper. Six most frequently used datasets are applied. The description about datasets is introduced in section 3.1. After that, relevant experiment settings are described in detail including data preprocessing in section 3.2, parameter estimation in section 3.3, and performance evaluation measures in section 3.4. Finally, in section 3.5, we introduce the experimental results and corresponding analysis.

### 3.1 Datasets

We conduct extensive experiments on six gene expression microarray datasets, and all of them are available online and most widely used in this field [2, 22]. Leukemia (ALLAML) and Colon can be downloaded from here: http://featureselection.asu.edu/datasets.php, Prostate can be downloaded from here: http://www2.cs.siu.edu/~qcheng/featureselection/, and the others can be downloaded from here: http://csse.szu.edu.cn/staff/zhuzx/Datasets.html. These datasets are all binary and almost all of them suffer from the problem of class imbalance. We choose binary datasets rather than multiclass ones because they are typical microarray dataset and more widely used in the published literature. The details are showed in Table 4. SDR denotes the sample-to-dimension ratio, i.e., (#class 1+#class 2) / #Features. IR stands for the imbalance ratio, which means #class 2 / #class 1.

**Table 4:** The characteristics of raw datasets.

Dataset	# Class 1	# Class 2	#Features	SDR	IR	Original Ref.
**Colon**	22	40	2000	3.1%	1.82	[23]
**CNS**	21	39	7129	0.84%	1.86	[24]
**Leukemia**	25	47	7129	1.01%	1.88	[25]
**Ovarian**	91	162	15154	1.67%	1.78	[26]
**Prostate**	59	77	12600	1.08%	1.31	[27]
**Breast**	46	51	24481	0.40%	1.11	[28]

To solve the class imbalance issue, The RVOS algorithm proposed in section 2.1 is applied to the six datasets mentioned above separately. Specially, new samples are obtained from class 1 so that #class 1 equals to #class 2. As the result, IR of all the datasets becomes 1.0 and SDR changes accordingly.

### 3.2 Data preprocessing

Empirically, we standardize each of the datasets (including the raw datasets and the balanced datasets) as zero mean and unit variance at first. Thus, the adverse effects caused by different genes with a huge gap in expression values are eliminated. Considering the mRMR method used in this paper is based on mutual information, it is necessary to discretize the datasets specially. When setting about to do that, we exploit the measure proposed in [12] as follows:

$$\tilde{x}=\left\{ \begin{array}{l} \text{+}2,\;\text{if}\;x\text{>}\mu \text{+}\sigma \text{/2}\\ -2,\;\text{if}\;x<\mu -\sigma \text{/2}\\ 0,\;\text{otherwise} \end{array}\right.$$

Where μ denotes the mean value and σ stands for the standard variance. $\tilde{x}=\text{+}2$ means over expression, $\tilde{x}=-2$ means under expression, and $\tilde{x}=0$ means the gene has normal expression.

Thus, we get two versions of datasets, discrete and continuous, which are all standardized. The discrete datasets are employed for mRMR while the continuous datasets are used for other feature selectors.

### 3.3 Parameter estimation

Before selecting features with LLSVM, SVM, Random Forest (RF) and classifying the transformed datasets with SVM, Naïve Bayes (NB), k-Nearest Neighbors (kNN) and Logistic Regression (LR), some parameters of them (except for NB) should be settled down first. For LLSVM, SVM and LR, the key parameter is the penalty factor C. The value of C affects the result of feature selection and the complexity of the classification model for SVM, LLSVM and LR. For RF, the depth is fixed as five, thus the number of basic trees becomes the vital parameter. Here we use N to represent the number of the basic trees, the larger N is the better performance will be obtained, in theory. But for a specific dataset, the effect becomes very limited when N exceeds a certain limit while the execution time of algorithm increases linearly. Therefore, we should set specific N values for different datasets. K refers the number of nearest neighbors to be selected for kNN. This value also requires patient tuning, too big or too small is not a good choice. When these models are employed as feature selectors or classifiers, we estimate the parameters with the corresponding model separately. Moreover, step size input (i.e., S) is another parameter need to be confirmed before applying VSSRFE with LLSVM or SVM (denoted as LLSVM-VSSRFE and SVM-VSSRFE respectively). In the process of specifying these parameters, we utilize stratified 5-fold cross-validation and grid search to achieve the best results. Table 5 shows the details. Most of the experiments in this paper are conducted on the balanced datasets, but in order to validate the performance of RVOS algorithm we tune C and S for SVM-VSSRFE on raw datasets. The details are presented in Table 6.

**Table 5:** Parameters for feature selectors and classifiers on balanced datasets.

		Parameter	Leukemia	Prostate	Ovarian	Breast	Colon	CNS
**Feature** **Selectors**	LLSVM	C	0.1	0.7	0.3	0.3	0.9	0.5
	SVM	C	0.1	0.9	0.5	0.1	0.1	0.05
	RF	N	100	800	100	400	200	400
**Step size**	LLSVM	S	600	1000	1000	800	100	200
	SVM	S	400	400	1000	800	200	100
**Classifiers**	SVM	C	9	1	3	0.09	7	0.1
	kNN	K	1	5	7	7	6	4
	LR	C	19	7	7	3	9	3

**Table 6:** Parameters for SVM-VSSRFE on raw datasets.

Feature selectors	Parameters	Leukemia	Prostate	Ovarian	Breast	Colon	CNS
**SVM**	C	0.3	0.9	0.5	0.1	0.5	0.9
**VSSRFE**	S	100	800	1000	1000	60	100

From Tables 5 and 6 we can see that the initial step size for different data sets is quite different. When the dataset have more genes (Breast, Prostate, Ovarian), the initial step size gets larger. On the contrary, the initial step size gets smaller when the dataset have fewer genes (Colon, CNS, Leukemia). This exactly confirms the assumption of gene importance and the basis for improving RFE described in section 2.2.

### 3.4 Performance evaluation measures

We choose three common measures as the performance evaluation measure in this study: Accuracy (ACC), area under ROC curve (AUC) and Matthew’s correlation coefficient (MCC). These measures are all widely used in classification evaluation task, among which, ACC and MCC are defined as follows:

$$\text{ACC}=\frac{TP\text{+}TN}{TP+FP+TN+FN}$$

$$MCC=\frac{TP\times TN-TP\times FN}{\sqrt{\left(TP+FP\right)\left(TP+FN\right)\left(TN+FP\right)\left(TN+FN\right)}}$$

TP denotes true positive, FP is false positive, TN is true negative and FN is false negative. ACC is the most common evaluation standard, and applying it alone is usually not enough. MCC is often chosen as one of the best final choice because even when the dataset is class imbalanced, MCC still can give back a good evaluation performance. MCC is essentially a correlation coefficient between the observation and predictive value, and its falls between +1 and -1. A coefficient of +1 represents a perfect prediction and -1 represents the worst. AUC takes into account both Ture Positive Rate (TPR) and False Positive Rate (FPR) whose definitions are as follows:

$$\text{TPR}=\frac{TP}{TP+FN}$$

$$\text{FPR}=\frac{FP}{FP+TN}$$

AUC can be seen as a probability value that one sample is classified correctly, the larger the better.

### 3.5 Results

In this section, we perform four sets of comparative experiments and a model evaluation experiment. In the first three sets of comparative experiments, we verify the proposed RVOS, VSSRFE and LLSVM algorithms respectively. And then, in the fourth set of experiments, we evaluate four common classifiers and discuss about which is more suitable as a classifier for microarray data. Finally, we conduct appropriate amount of experiments to evaluate the generalization capability of the classification model.

What have to be highlighted is that all the experiments are conducted with stratified 5-fold cross-validation. We opt for 5-fold cross-validation because it is a common choice in this domain, and the stratified cross-validation strategy guarantees that the proportions of instances belonging to two classes both in the training set and test set are equal. The experiments in this literature are founded on two public machine learning libraries called scikit-learn and scikit-feature [29], which are open source and accessible online. The former has gone through more than one release, and we decide to use the latest stable version 0.18. Readers can find it from this website: http://scikit-learn.org/stable/index.html.

#### 3.5.1 Comparative experiments of datasets balanced with RVOS and raw datasets

In this section, we balance the datasets with RVOS and then conduct a set of experiments with SVM-VSSRFE on six raw and balanced datasets to select genes. We choose SVM-VSSRFE as the feature selector because SVM-RFE is very time-consuming, and SVM-VSSRFE can achieve the same goal more quickly. Linear SVM (C = 1) is chose as the classifier, and each dataset is executed 128 times to select 1 to 128 genes, separately.

**Fig 1:**
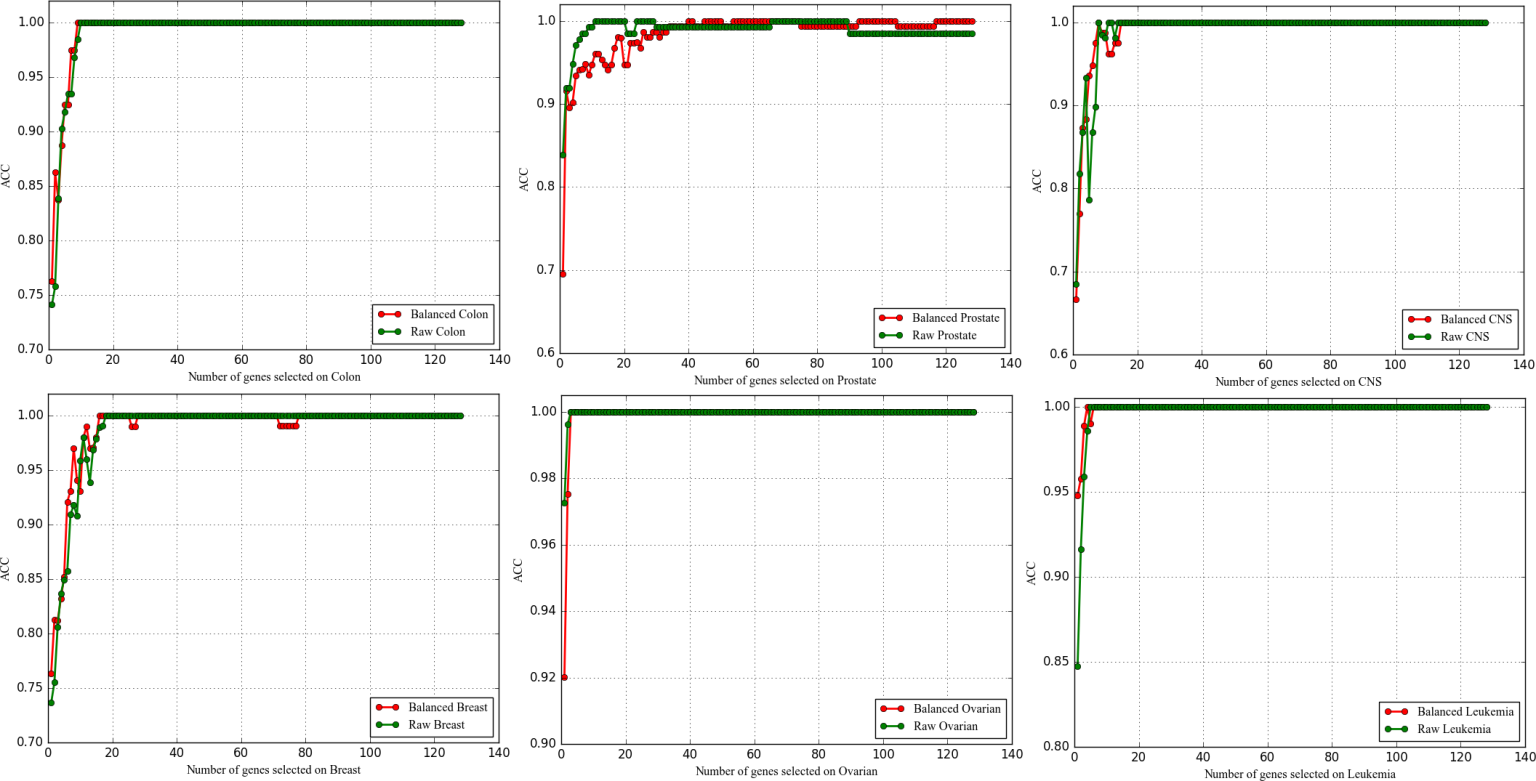
The comparison of ACC obtained on six balanced and raw datasets.

**Fig 2:**
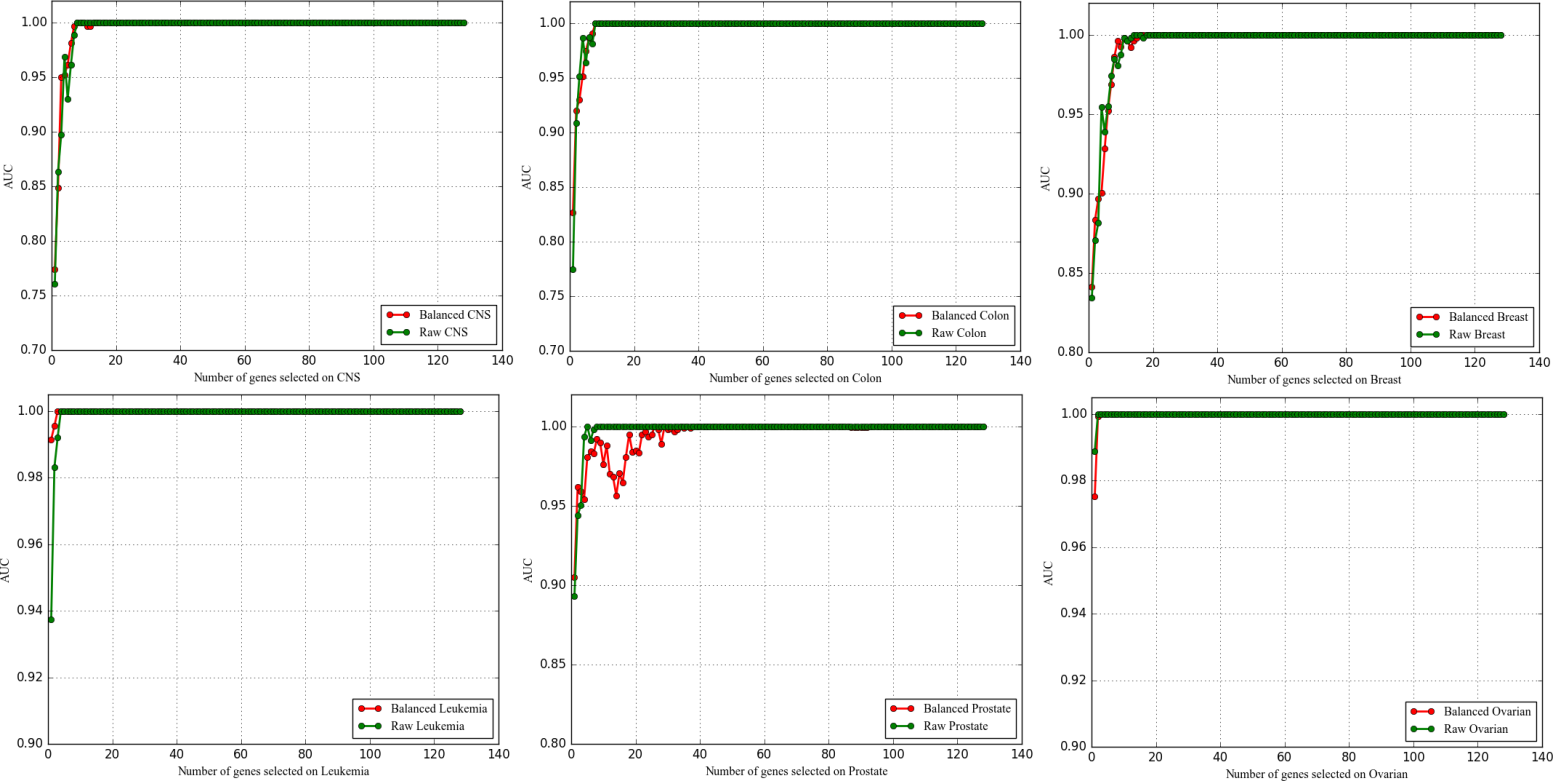
The comparison of AUC obtained on six balanced and raw datasets.

**Fig 3:**
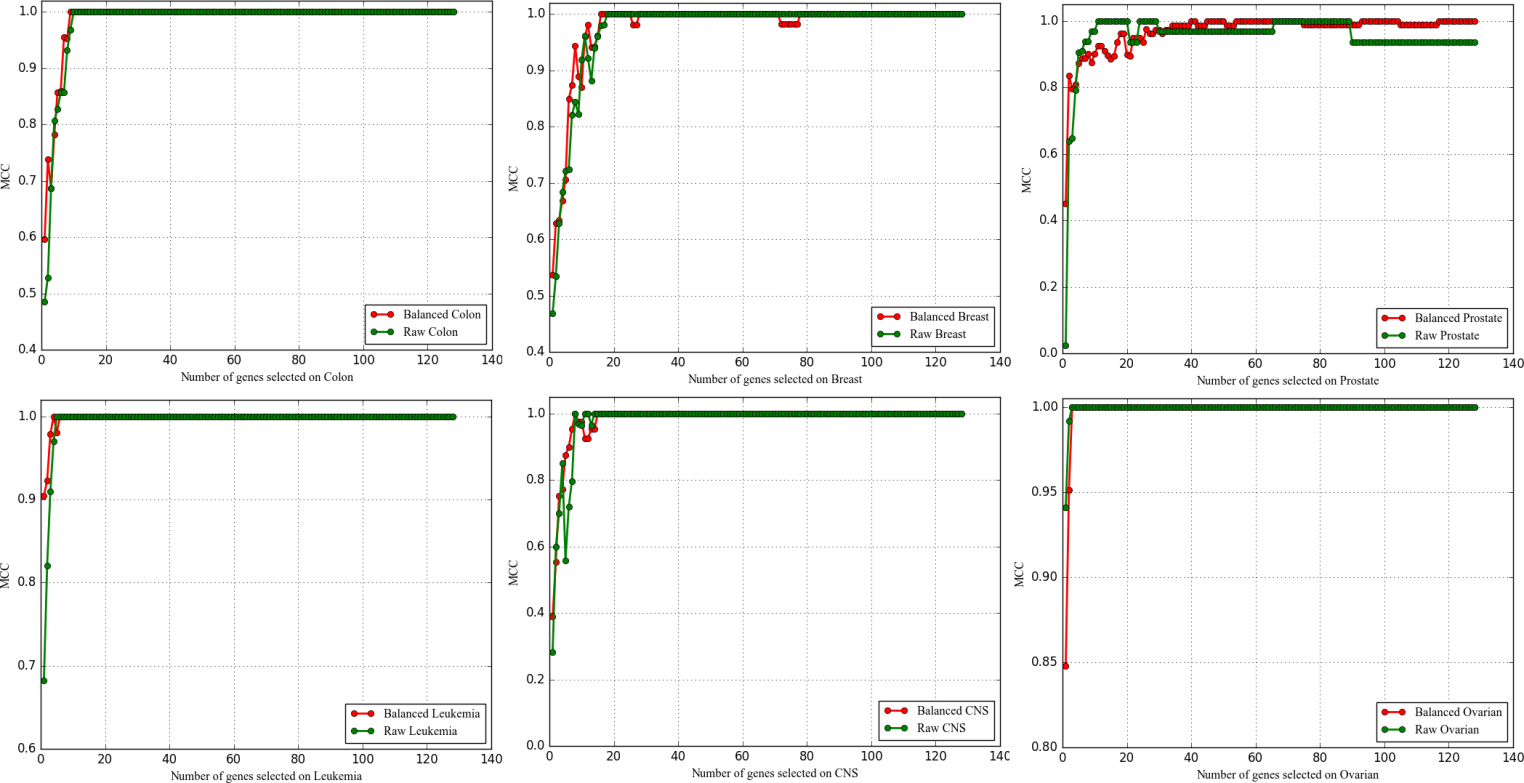
The comparison of MCC obtained on six balanced and raw datasets.

Figs 1–3 represent the performance comparison of raw and balanced datasets on three evaluation measures respectively, from which we can see that the balanced CNS and Leukemia obtain better performances on all the evaluation measures; the balanced Breast and Colon outperform on ACC and MCC while they are comparable on AUC. This indicates that the RVOS is indeed a good choice to try to solve the class imbalance problem for microarray data in the future.

And, of course, we observe that the performances of the balanced Ovarian and Prostate are unsatisfactory, but this occurs when fewer genes are selected. As the number of genes increase, the results achieved by the balanced datasets get equal to or better than the raw datasets. In addition, it is confusing that some raw datasets with higher IR value achieve better results after balanced (CNS, Colon, Leukemia) while the lower one gets worse (only Prostate, Breast gets better performance). Considering the randomness of the new sample generated by the RVOS algorithm and the complexity of the gene expression data, we consider this to be the result of noise interference [10]. Beyond that, the particularity of the dataset itself may be another considerable factor that leads to this situation. The RVOS can be seen as a data preprocessing method in this study, and the following experiments are carried out based on this.

#### 3.5.2 Comparative experiments of VSSRFE and RFE

This section aims to validate the effectiveness of VSSRFE. We use traditional linear SVM as the basic feature selector, and combine it with RFE and VSSRFE separately to conduct the experiments on the six balanced datasets. Two group experiments are conducted under the same conditions except the step size of recursive feature elimination: one is fixed to one, and the other is determined by the input initial value and the number of features to be eliminated. In addition to this, we choose four as the number of genes to select because four is relatively small, as some researchers doubted, which is likely to get an adverse effect on the quality of feature selection. Also, linear SVM (C = 1) is chose as the classifier.

Table 7 shows the evaluation results and time consumption of SVM-RFE and SVM-VSSRFE. As seen, the time consumption is greatly reduced by using VSSRFE on all the six balanced datasets, and most of the datasets are reduced by hundreds times. Interestingly, SVM-VSSRFE has gained more time consumption reduction on higher dimensional datasets (Breast, Ovarian, Prostate) and less time consumption reduction on most of the lower dimensional datasets (CNS, Colon). We then can conclude that VSSRFE has a better effect on super high dimensional data. From this point of view, VSSRFE does work well and provides a good idea for efficient feature selection in this field. On the other hand, compared with the RFE, VSSRFE decreases the performance to some extent on three datasets while obtaining better results on the other three datasets (the best performances are outlined in bold face). The decreased performance seems to suggest that the change of the step size does affect the quality of feature selection just as other researchers worried. However, this does not negate the effect of the variable step size itself. Section 2.2 has explained that the feature selection process of VSSRFE is a rough to careful process. The decline of classification accuracy on some datasets should be attributed to the radical update conditions and update rule of VSSRFE's step size. In fact, the “change” of the step size can be changed in many ways. For example, the update condition in Table 2 can be replaced by “If temp / N = 1.5 and S > 1:” and the update rule also can be changed as “S = S / 3;” and so on. On the contrary, more radical update conditions and update rule can be tried on the datasets that have achieved higher classification accuracy. Perhaps such attempts can obtain faster and better results. All of these flexible adjustments may bring a great reduction of time consumption on the basis of trying not to affect the quality of feature selection. We do not do this in depth, but there are reasons to believe that this is feasible.

**Table 7:** The comparison of performance and time consumption between SVM-RFE and SVM-VSSRFE.

	SVM-RFE				SVM-VSSRFE			
	ACC	AUC	MCC	Time (s)	ACC	AUC	MCC	Time (s)
**Prostate**	**0.9220**	**0.9846**	**0.8509**	10468.94	0.9021	0.9538	0.8096	**37.82**
**Breast**	**0.8609**	**0.9447**	**0.7284**	20518.44	0.8318	0.9003	0.6685	**35.88**
**CNS**	0.7696	0.8765	0.5504	1009.01	**0.8839**	**0.9522**	**0.7725**	**14.57**
**Colon**	**0.9375**	**0.9875**	**0.8764**	76.69	0.8875	0.9516	0.7820	**0.69**
**Ovarian**	0.99	**1.0**	0.9809	1435.81	**1.0**	**1.0**	**1.0**	**4.77**
**leukemia**	**1.0**	**1.0**	**1.0**	13897.13	**1.0**	**1.0**	**1.0**	**18.96**

#### 3.5.3 Comparative experiments of LLSVM-VSSRFE and the other four typical feature selectors

This section is intended to verify the efficiency of LLSVM. LLSVM combining VSSRFE as a feature selector is compared with SVM-VSSRFE, random forest (RF), mRMR and relief [30]. The main reason why we choose SVM-VSSRFE instead of SVM-RFE is that the latter is too time-consuming to bear. The same as the experiments introduced above, linear SVM (C = 1) is used as the classifier and each balanced dataset is executed 128 times to select 1 to 128 genes separately.

Table 8 shows the time consumption of LLSVM-VSSRFE and SVM-VSSRFE, from which we can see that the time consumed by LLSVM-VSSRFE is reduced greatly (The best performances are outlined in bold face), especially when the dimensions are particularly high (e.g., Prostate, Breast). This indicates that the training speed of LLSVM is much faster than that of traditional SVM, and combined with VSSRFE makes the performance better.

**Table 8:** The comparison of time consumption (s) between SVM-VSSRFE and LLSVM-VSSRFE.

	SVM-VSSRFE	LLSVM-VSSRFE
**Prostate**	4775.05	**325.25**
**Breast**	4541.82	**970.16**
**CNS**	1890.59	**355.90**
**Colon**	**96.97**	100.04
**Ovarian**	2256.16	**650.58**
**Leukemia**	741.62	**97.08**

**Fig 4:**
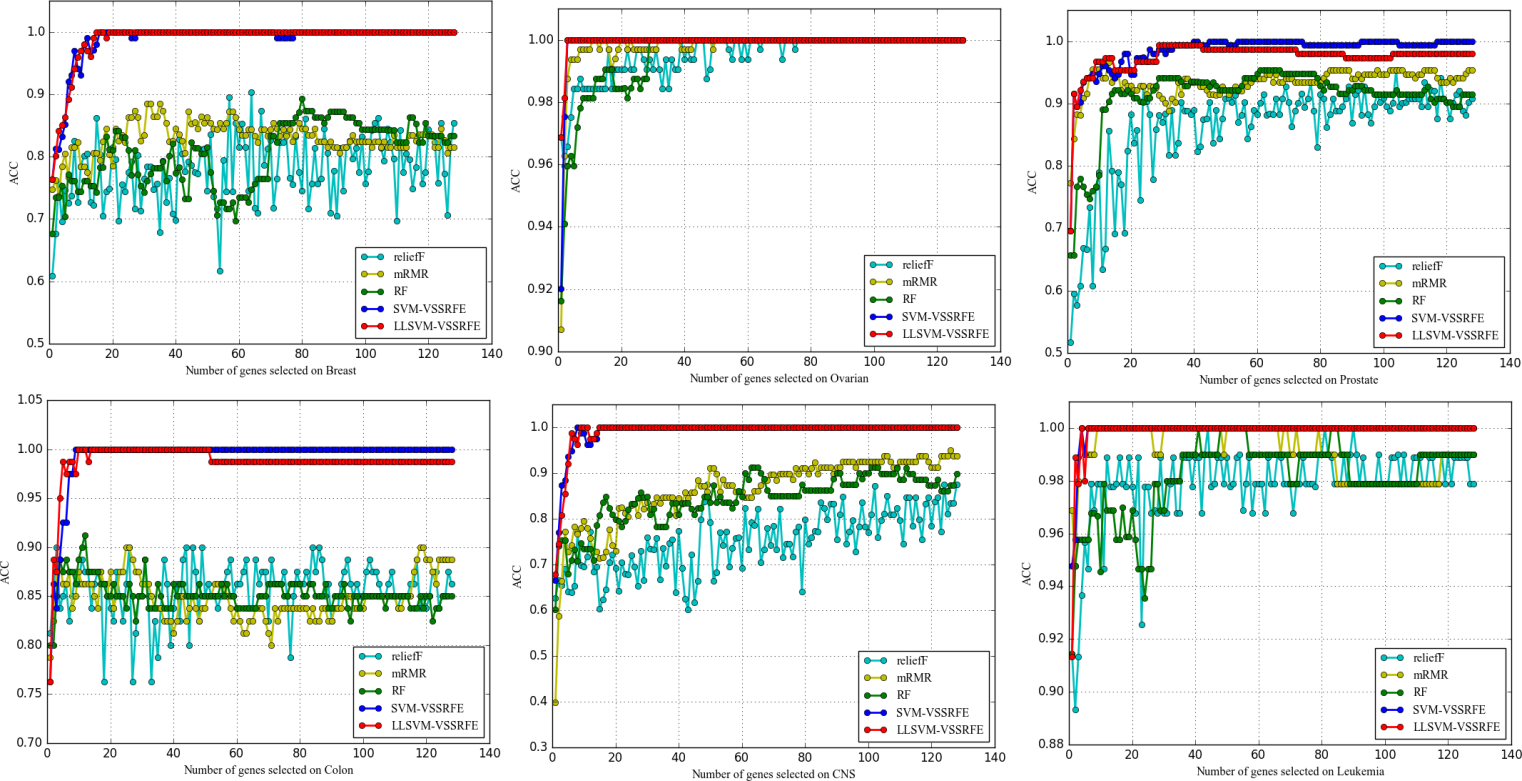
The comparison of ACC obtained by five feature selectors.

**Fig 5:**
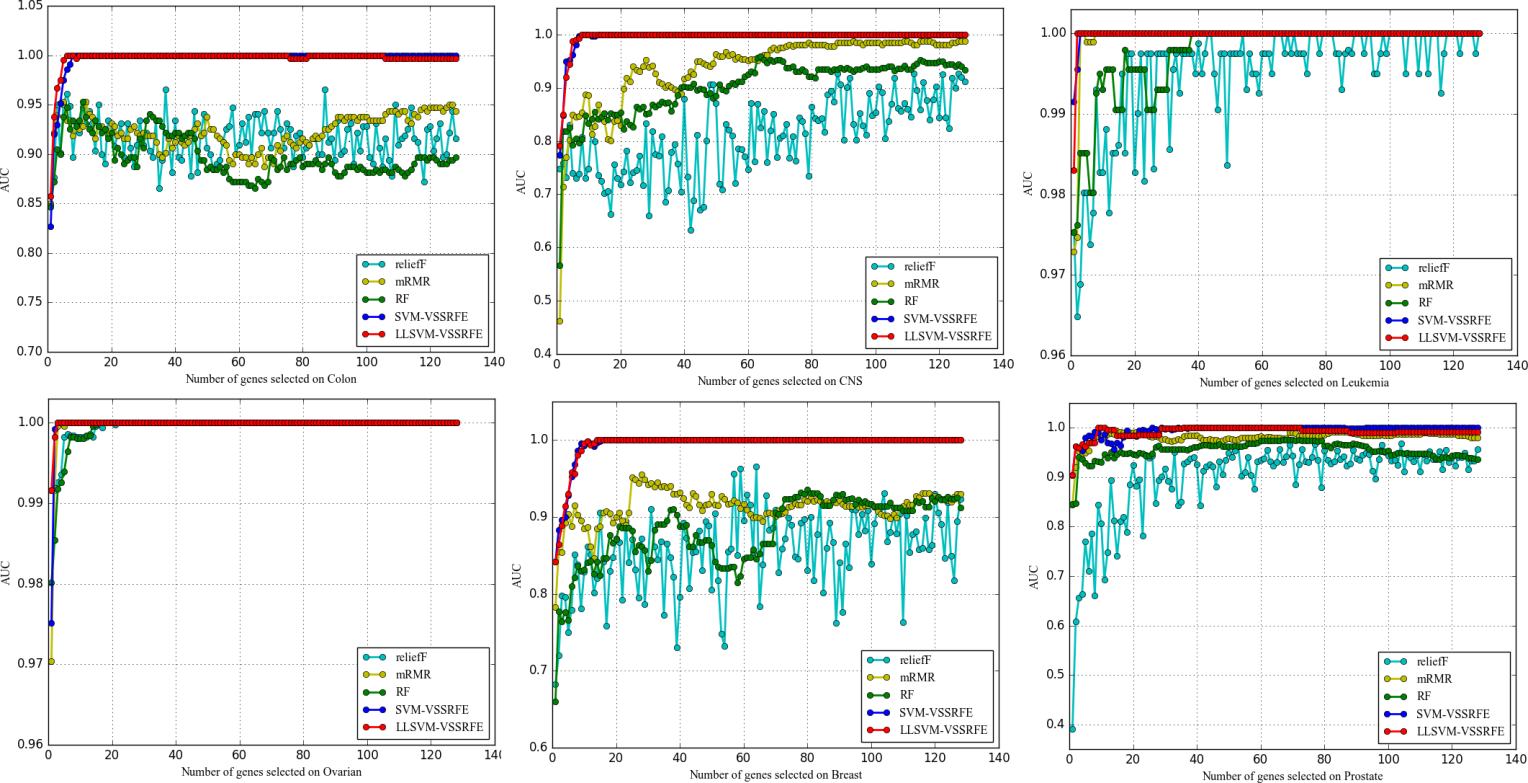
The comparison of AUC obtained by five feature selectors.

**Fig 6:**
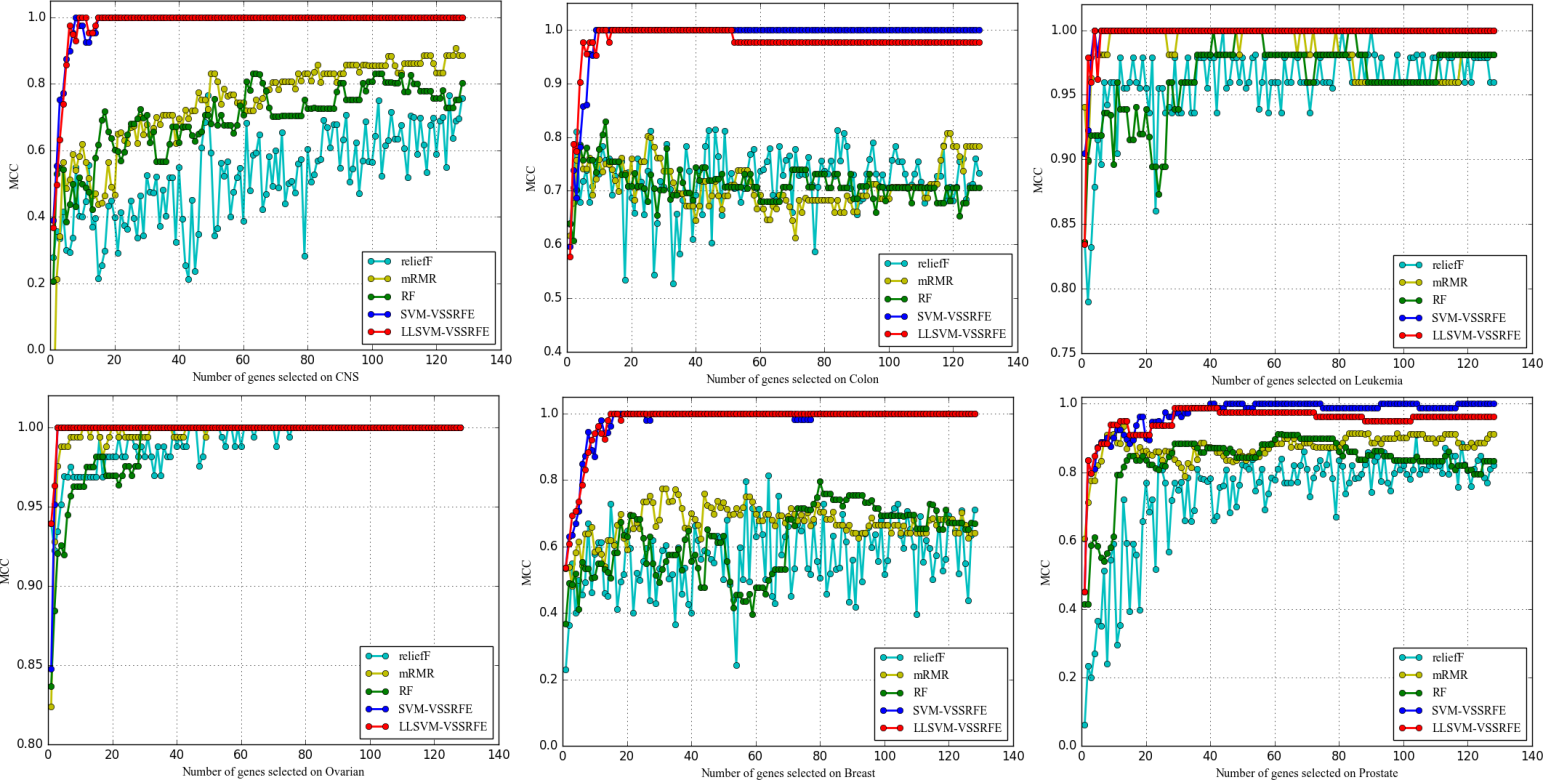
The comparison of MCC obtained by five feature selectors.

Figs 4–6 show the feature selection quality of five feature selectors. From the figures we can observe that the curves of reliefF on six datasets are the most unstable, and the values of three evaluation measures on some datasets (Breast, CNS, Leukemia, Prostate) are the lowest. Compared with reliefF, the curves of mRMR and RF are more stable, but their evaluation values are much lower compared to SVM-VSSRFE and LLSVM-VSSRFE. To be more specific, both SVM-VSSRFE and LLSVM-VSSRFE can make the classifier's evaluation values be 100% when selecting 8 genes or fewer from most of the datasets (except for Breast and Prostate). As far as SVM-VSSRFE and LLSVM-VSSRFE are concerned, LLSVM-VSSRFE works equally or better in terms of Breast, CNS, Leukemia, and Ovarian. In cases of Colon and Prostate, LLSVM-VSSRFE outperforms when fewer genes are selected whereas SVM-VSSRFE is the opposite. To sum up, LLSVM-VSSRFE has obtained comparable results both in feature selection efficiency and feature selection quality, especially in feature selection efficiency. That is to say, increasing the step size appropriately will not bring negative effects on the quality of feature selection. On the contrary, it sometimes can even achieve higher classification accuracy for microarray data. With the growth of DNA microarray data, we believe that LLSVM-VSSRFE will play a significant role in the future.

#### 3.5.4 Comparative study of four common classifiers

In this section, we carefully validate four typical classifiers, including Naïve Bayes (NB), k-Nearest Neighbors (kNN), linear SVM (SVM) and L2 regularized Logistic Regression (LR). LLSVM-VSSRFE is employed as the feature selector to select 1 to 32 genes from the balanced datasets and then evaluate these genes with these well-tuned classifiers. In addition, we utilize LLSVM-VSSRFE as the feature selector and LR as the classifier to conduct experiments on three balanced datasets, which is aimed to get the training scores and testing scores so as to evaluate the generalization capability of the classification model.

**Fig 7:**
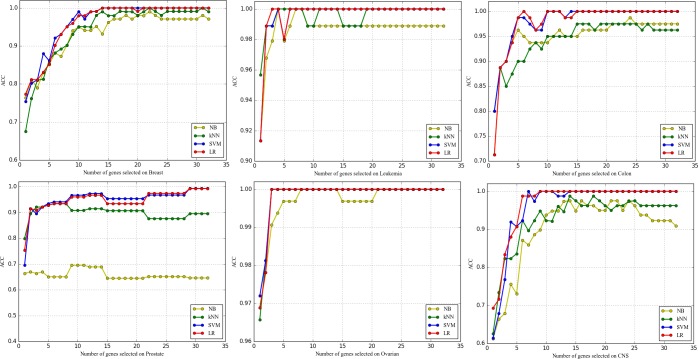
The comparison of ACC obtained by four common classifiers.

**Fig 8:**
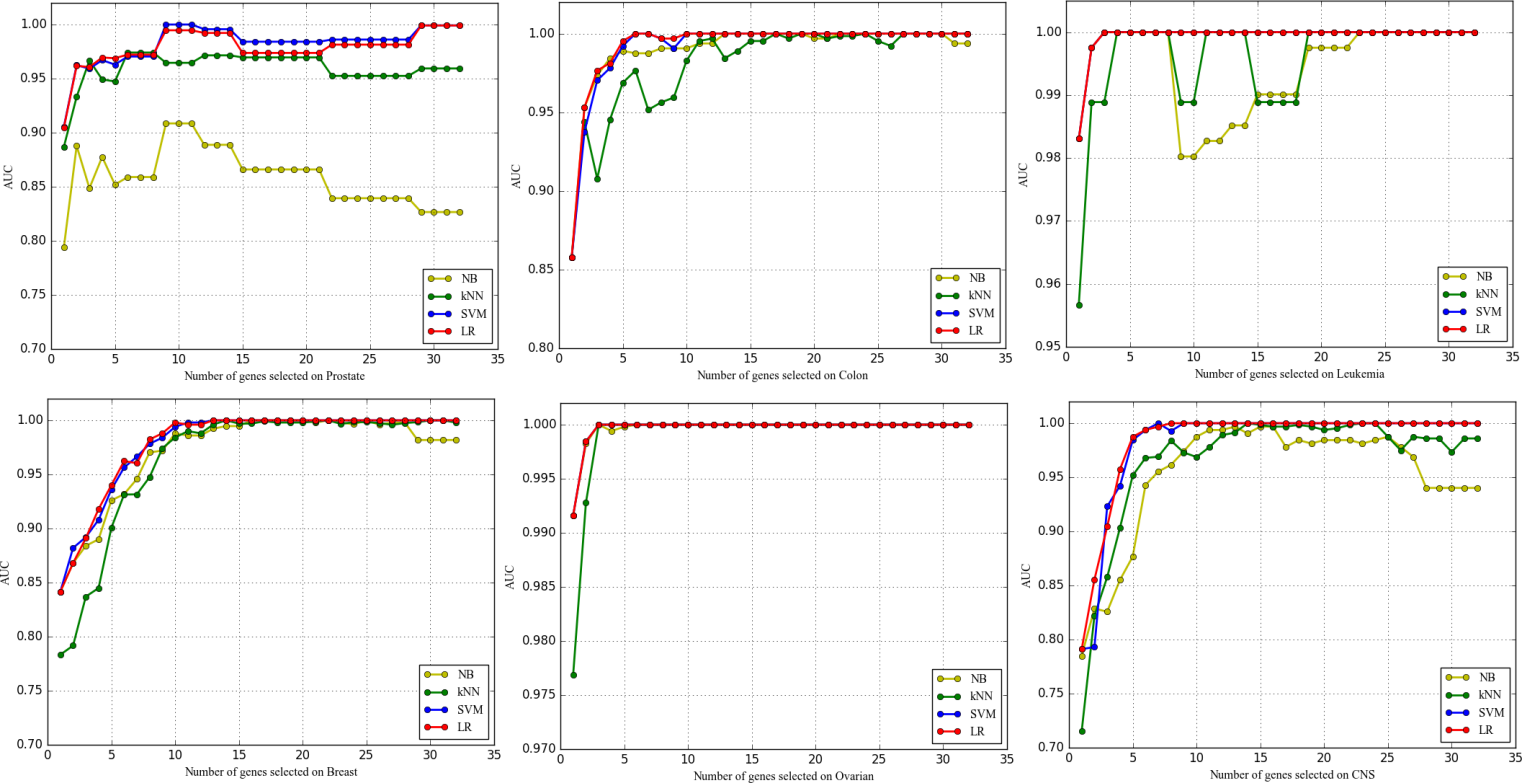
The comparison of AUC obtained by four common classifiers.

**Fig 9:**
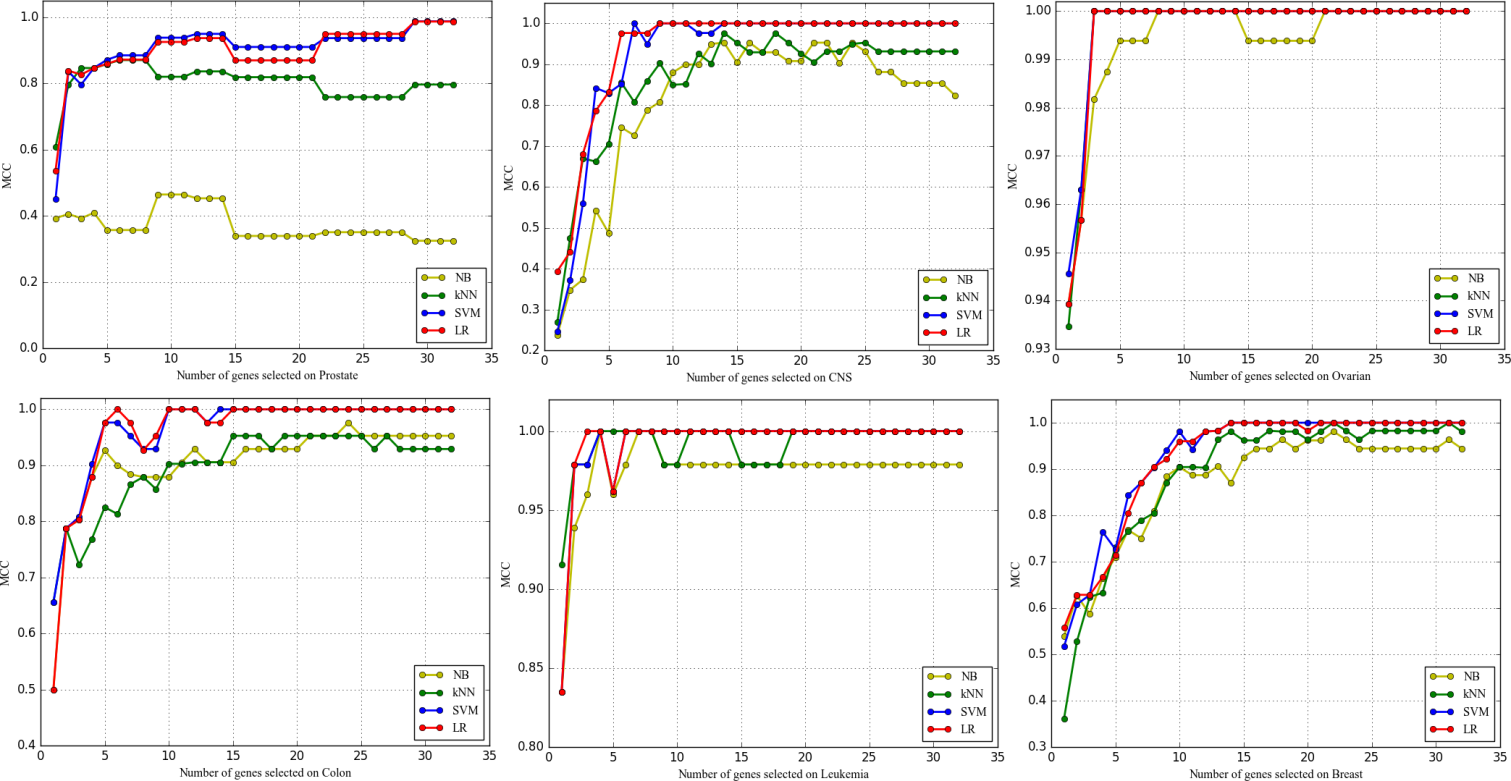
The comparison of MCC obtained by four common classifiers.

Figs 7–9 show the effects of classifiers on the classification performance. As we can see, the results obtained by different classifiers on the same dataset can be very different, and SVM and LR outperform the three evaluation measures over all the datasets. For microarray data, the expression value of each gene has many variations even if the samples belong to the same category. That is disadvantageous for both NB and kNN, Because NB is good at separating samples with fewer feature values and KNN is directly affected by the distance between the sample points which is determined by the feature values. On the contrary, Microarray data is linearly separable, so it is especially suitable for linear models such as SVM and LR.

Concretely, for SVM and LR, SVM has few advantages (except for the Breast and Prostate on some of the evaluation measures). This is quite surprising, because SVM is always regarded as the best classifier for the existing research in this field. In addition, the additional information we can observe is that the curves of LR are smoother than that of SVM. That is to say, the performance of LR is more stable. Most importantly, LR is a simple classifier and easier to implement which means much for dataset with too little sample size just like microarray data. Comprehensively, as a classifier for microarray data analysis, Logical Regression should be paid more attention for the scientific community.

**Fig 10:**
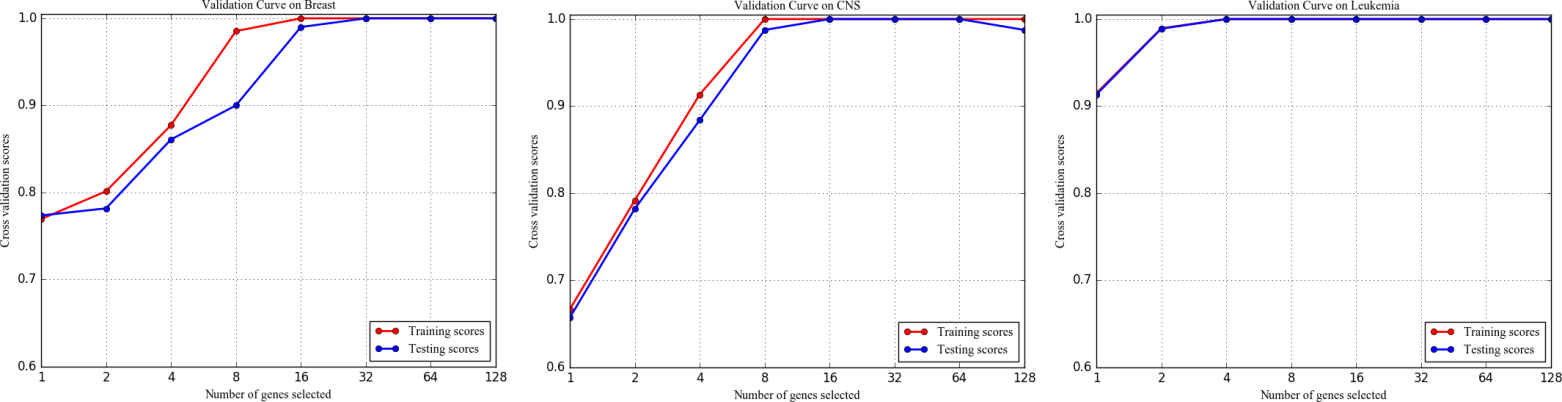
The classification model evaluation.

Fig 10 shows the results of the classification model evaluation when 1, 2, 4, 8, 16, 32, 64, 128 genes are selected respectively. As seen, testing scores are very close to the training scores, especially when more genes are selected. This indicates that the classification model has good generalization [31].

#### 3.5.5 Discussion

Although the proposed methods in this paper have achieved promising results compared with the existing methods, there are still some topics should be discussed in depth:

(1) The methods proposed in this paper are implemented based on two Python libraries, but the execution efficiency of Python is not the fastest. So our approach can be further optimized, such as utilizing C or C++ to encode and implement.
(2) Although experimental results show that RVOS has achieved good performance, the algorithm also has some limitations. If the number of samples belonging to the rare class is quite small and many more supplementary samples are needed, RVOS may not work well as in this paper because the number of expression values for each gene is limited. To solve this problem, much more hard research work needs to be done in the future.
(3) A main shortcoming for VSSRFE strategy is to choose the best initial value of step size, which is related to dataset, update condition and rule of the step size. What’s worse, in some cases, the selection of the best initial value of step size is depended on the number of genes to select. This means that some adjustment work is necessary in order to determine this parameter. Fortunately, VSSRFE is efficient enough, so the related adjustment work is acceptable to some extent just as the k for KNN. In addition, the update conditions and rule of step size can be adjusted flexibly, which will have a direct impact on the efficiency of the feature selection process and the quality of the feature selection results. If the update conditions and rule of step size are set reasonably, the results will get better.
(4) Because of the characteristics of microarray data, more advanced analytical techniques are required. In recent years, representation-based methods are of great interest both for feature selection [32, 33] and classification [34, 35], especially in the field of image processing and computer vision. These methods can be introduced for microarray data analysis for further research.
(5) This article does not pay attention to the relationships between genes (features). The existing studies have shown that there are some relationships or effects between genes, but the identification of gene interaction is a rather complex issue, especially for researchers who have no biomedical background. This paper focuses on the efficient screening of key genes and the classification of microarray data. The interaction recognition between genes can be further studied in the future.

## 4. Conclusion

Complex diseases such as breast cancer remain the greatest threat to human life. The growth of microarray data and the development of statistical methods have provided new possibilities for the prediction and treatment of such diseases. Feature selection and classification are the core technologies of microarray data analysis. They both play key roles in genes recognition and diseases diagnosis. Limited to the characteristics of microarray data, many typical methods in this field still need to be paid more attentions to overcome their disadvantages.

Small sample size, high dimensionality and class imbalance are the main characteristics of microarray data as well as the main challenges for researchers to conduct microarray data analysis. Among them, Class imbalance is rarely studied by researchers in this field. To preprocess the datasets, this paper firstly proposes a simple but effective resampling method called RVOS to solve this issue. By doing this, the distribution of two kinds of samples is balanced and the problem of small sample size is also alleviated to some extent.

SVM-RFE is a typical method which is widely studied by researchers in this field. To reduce the time consumption of SVM-RFE Fundamentally, we firstly propose an improved version of RFE called VSSRFE. VSSRFE tries to reduce the recursion times by a large step size, and keep the step size decreasing while the number of features to be eliminated is getting smaller and by this way to ensure the quality of the meaningful genes selected. There are thousands of genes in human beings, so it means much to apply an efficient feature selection strategy. VSSRFE provides an interesting idea to speed up the procedure of gene selection. This may contribute to the microarray data analysis in the future. Moreover, we induce another efficient implementation of linear SVM called LLSVM. LLSVM is a kind of pure linear classifier based on support vector, so it inherits the advantages of SVM and reduces unnecessary computational cost for large-scale linear separable data such as microarray data. Combined with VSSRFE, LLSVM-VSSRFE becomes an efficient and effective feature selector compared with the existing methods and has potential in the gene selection field.

Finally, we conduct a study on the effects of different classifiers on the classification results and observe that sometimes L2 regularized Logistic Regression is a better choice for microarray data classification. This is also a question that worth paying more attention to validate in the future.

## Supporting information

S1 File dataPreprocess. Section 3.2 and section 3.5.1 are implemented by this code file.
(PY) Click here for additional data file.

S2 File VSSRFE&RFE. Section 3.5.2 is implemented by this code file.
(PY) Click here for additional data file.

S3 File LLSVM-VSSRFE. This code file implements LLSVM-VSSRFE and is used to conduct experiments in section 3.5.3.
(PY) Click here for additional data file.

S4 File LLSVM&SVM. This code file implements LLSVM-VSSRFE and SVM-VSSRFE and is used to conduct experiments in section 3.5.3.
(PY) Click here for additional data file.

S5 File mRMR. **This** code file implements mRMR and is used to conduct experiments in section 3.5.3.
(PY) Click here for additional data file.

S6 File randomForest. This code file implements random forest and is used to conduct experiments in section 3.5.3.
(PY) Click here for additional data file.

S7 File reliefF. This code file implements reliefF and is used to conduct experiments in section 3.5.3.
(PY) Click here for additional data file.

S8 File SVM-RFE. This code file implements SVM-RFE and is used to conduct experiments in section 3.5.2.
(PY) Click here for additional data file.

S9 File classifiers. Section 3.5.4 is implemented by this code file.
(PY) Click here for additional data file.

S10 File modelEvaluation. Section 3.5.5 is implemented by this code file.
(PY) Click here for additional data file.

S11 File Breast. (ARFF)

S12 File CNS. (ARFF)

S13 File Colon. (MAT)

S14 File Leukemia. (ARFF)

S15 File Ovarian. (ARFF)

S16 File prostate_TumorVSNormal_test. (CSV)

S17 File prostate_TumorVSNormal_train. (CSV)
